# National Implementation of a Tuberculosis Sample Referral Network to Expand Access to Molecular Diagnosis in the Republic of Congo: Pilot Evaluation and Programmatic Analysis (2023–2025)

**DOI:** 10.3390/tropicalmed11080229

**Published:** 2026-08-17

**Authors:** Darrel Ornelle Elion Assiana, Hugues Check Asken Traoré, Franck Hardain Okemba-Okombi, Emmanuel Fonfon Ebata-Mboussa, Freisnel Hermeland Mouzinga, Erudit Beny Elenga, Asta-Wabi Fozia Hamed Belo, Mary Vincent Pabie Passy Mambou, Juliette Burchelle Ayessa Ondzie, Alain Disu Kamalandua, Tanou Joseph Kalivogui, Romeance Juvick Lepoupou, Bermeland Ewuinh Tsiobinda, Edrene Likoyo Mampouyath, Rachel Laure Nguela, Viviane Gisele Lompo Ouedraogo, Prudence Théoline Ovoulaka, Jessica Jeielle Ayande, Jacques Ndion Ngandzien, Baurel Arnaud Akiera, Salomon Tchuandom Bonsi, Jean Akiana

**Affiliations:** 1 Faculté des Sciences et Techniques, Université Marien Ngouabi, Brazzaville P.O. Box 69, Congo; freisnelm@gmail.com (F.H.M.); 23akianajean@gmail.com (J.A.); 2 Laboratoire National de Référence des Mycobactéries, Brazzaville P.O. Box 1066, Congo; franckokemba@gmail.com (F.H.O.-O.); emmanuelebata08@gmail.com (E.F.E.-M.); elengabeny3001@gmail.com (E.B.E.); faouziab521@gmail.com (A.-W.F.H.B.); paolapassy6@gmail.com (M.V.P.P.M.); julieayessa2@gmail.com (J.B.A.O.); salomon.tchuandom.bonsi@undp.org (S.T.B.); 3 Programme National de Lutte contre la Tuberculose, Brazzaville P.O. Box 1066, Congo; prudencetheolineovoulaka@gmail.com (P.T.O.); ayandejeielle@gmail.com (J.J.A.); asnathndion@gmail.com (J.N.N.); baurelakiera@gmail.com (B.A.A.); 4 United Nations Development Programme, Brazzaville P.O. Box 465, Congo; hugues.traore1@undp.org (H.C.A.T.); alain.disu.kamalandua@undp.org (A.D.K.); tanou.joseph.kalivogui@undp.org (T.J.K.); juvick.lepoupou@undp.org (R.J.L.); bermeland.ewuinh.tsiobinda@undp.org (B.E.T.); edrene.liyoko.mampouyath@undp.org (E.L.M.); rachellaure.nguela@undp.org (R.L.N.); viviane.gisele.lompo.ouedraogo@undp.org (V.G.L.O.); 5 Department of Medicine and Health Services Research, University of Heidelberg, D-69047 Heidelberg, Germany; 6 Faculté des Sciences de la Santé, Université Marien Ngouabi, Brazzaville P.O. Box 2672, Congo; 7 Service de Pneumologie, Centre Hospitalier Universitaire, Brazzaville P.O. Box 32, Congo; 8 Fondation Congolaise pour la Recherche Médicale, Brazzaville P.O. Box 2672, Congo; 9 Direction des Technologies de la Santé, Ministère de la Santé et de la Population, Brazzaville P.O. Box 1066, Congo

**Keywords:** tuberculosis, rifampicin resistance, sample referral, Xpert MTB/RIF Ultra, national scale-up, Republic of Congo

## Abstract

Background: Limited access to rapid molecular diagnostics remains a major barrier to timely tuberculosis (TB) detection and drug-resistance surveillance in resource-constrained settings. In the Republic of Congo, GeneXpert MTB/RIF platforms remain concentrated in a few laboratories, while peripheral facilities rely predominantly on smear microscopy, highlighting the need for a structured sample referral system. Methods: We conducted a three-year mixed-methods operational study comprising a six-month pilot phase (January–June 2023), initially enrolling 29 TB diagnostic and treatment centers in urban and rural settings followed by a 30-month national roll-out phase (July 2023–December 2025), evaluated retrospectively using routine programmatic surveillance data. Results: During the pilot phase, 962 sputum samples were transported. 294 (30.6%) were TB cases, including 12 (4.1%) RR-TB. Median total turnaround time was 53 h (19–168) in urban and 62 h (26–204) in rural, with 86% overall site participation (92% urban vs. 60% rural). During scale-up, samples increased from 2154 (2023) to 5160 (2024), achieving full national coverage (12/12 departments). Over three years, 2587 TB and 84 RR-TB cases were detected. The system’s contribution to national TB detection grew from 9.2% to 16.0%, and RR-TB detection from 10.4% to 18.7%. Conclusions: Implementation of the structured sample referral system was associated with improved equitable access to molecular TB diagnosis, turnaround times within national targets, and strengthened the RR-TB surveillance. Scalable transport networks integrated into national guidelines may enhance diagnostic equity and drug-resistance detection in centralized laboratory systems.

## 1. Introduction

Tuberculosis (TB) remains a leading cause of morbidity and mortality from infectious diseases globally, particularly in low-resource settings. Globally, the estimated TB incidence and mortality rate is approximately 131 and 15 per 100,000 population, respectively, reflecting ongoing transmission and the persistent public health burden of TB [1].

In the Republic of Congo, TB continues to pose a major public health challenge, with an estimated incidence and mortality of 302 and 40 cases per 100,000 population, respectively. Despite substantial progress in diagnostic and treatment capacity, including the establishment of a national reference laboratory for mycobacteria in 2018 and its progressive operationalization through 2019–2022, access to rapid molecular diagnostics remains limited in peripheral health facilities [1,2,3].

Although GeneXpert testing has been introduced as the initial diagnostic assay in equipped centers, many diagnostic and treatment centers still rely primarily on microscopy. To address this gap, a structured sample referral system linking peripheral collection sites to GeneXpert equipped laboratories was implemented [3].

Sample referral systems, as recommended by the World Health Organization (WHO) in its consolidated TB diagnosis guidelines and molecular testing policies, are essential to extend access to WHO-recommended diagnostic tools such as Xpert MTB/RIF Ultra. These systems ensure that samples collected at peripheral sites can be safely transported and tested in centralized laboratories with rapid turnaround and reliable reporting, thereby overcoming access gaps and reducing diagnostic delay [4,5,6]. However, empirical evidence or peer-reviewed data evaluating the operational performance and national-level impact of sample referral systems in Central African settings remain scarce. While implementation experiences have been reported across sub-Saharan Africa, the existing literature predominantly documents pilot implementations of integrated (Ghana) or hub-and-spoke referral models (Uganda), district-level evaluations of Xpert MTB/RIF Ultra deployment, cost-effectiveness and decentralization modelling analyses (Mozambique and Tanzania), and turnaround time assessments conducted in specific provinces or programmatic contexts (South Africa). These settings differ from the Republic of Congo in laboratory density and transport infrastructure, limiting direct applicability of their findings to the Congolese context. Referral-based drug-resistance surveillance systems have been described in Central Africa, including national RR-TB referral networks in Gabon and the Central African Republic; however, longitudinal analyses quantifying the proportional contribution of such referral/transport systems to national-level TB and rifampicin-resistant TB case detection over time remain scarce in this region [7,8,9,10,11,12].

Referral-based drug-resistance surveillance systems have been described in Central Africa, including national RR-TB referral networks in Gabon and the Central African Republic however, longitudinal analyses quantifying the proportional contribution of such referral/transport systems to national-level TB and rifampicin-resistant TB case detection over time remain scarce in this region [13,14].

The Republic of Congo has a population of approximately 6.1 million, with more than half of its inhabitants concentrated in the two largest urban centres, Brazzaville (2.1 million) and Pointe-Noire (1.4 million), while the remaining ten departments are comparatively rural and sparsely populated. Tuberculosis (TB) control is implemented through a vertical National Tuberculosis Control Programme (PNLT).

Although molecular TB diagnostic capacity has expanded in recent years, the health system remains largely centralized, with GeneXpert platforms concentrated in a limited number of urban reference laboratories. Consequently, peripheral health facilities throughout the country’s 12 departments have historically relied primarily on sputum smear microscopy for TB diagnosis. Prior to this intervention, no standardized mechanism existed to connect these peripheral sites with centralized molecular testing services, resulting in delayed diagnosis, under-detection of rifampicin resistance TB, and weak integration between laboratory services and routine programmatic reporting.

To address this gap, the National Tuberculosis Control Program of the Republic of Congo piloted a sample referral system to assess feasibility, operational performance, and diagnostic timeliness. Following favorable pilot findings, the system was progressively expanded nationwide. This study describes the implementation of the national sample referral system, presents results from the pilot phase (January–June 2023), and documents outcomes of the subsequent national roll-out using routine programmatic data (July 2023–December 2025), with particular emphasis on its contribution to national TB and rifampicin-resistant TB detection. The primary objective of this study was to evaluate the contribution of the national sample referral system to national TB and rifampicin-resistant TB case detection over a three-year period. Secondary objectives were to assess the system’s operational feasibility, defined as the proportion of selected sites actively participating throughout the pilot phase, and to document its performance in terms of turnaround time and diagnostic yield across both the pilot and national roll-out phases.

## 2. Methods

### 2.1. Study Design

This was a descriptive implementation research study evaluating the operational feasibility, performance, and scale-up of a sample referral system for tuberculosis diagnosis in the Republic of Congo. The study comprised two phases:

✓ Pilot phase (January–June 2023): Prospective operational assessment in selected sites with systematic documentation of turnaround times, participation rates, diagnostic yield, and implementation barriers.
✓ National roll-out phase (July 2023–December 2025): Progressive geographic expansion with programmatic monitoring of network growth, sample transport volumes, and contribution to national TB case detection.

This was an implementation evaluation. The focus was on describing system performance under real-world conditions, identifying operational bottlenecks, and documenting scalability.

### 2.2. Study Setting

Geographic scope: Republic of Congo, 12 departments.

Health system context: National Tuberculosis Control Program with an established network of TB Diagnostic and Treatment Centres (CDTs; French: *Centres de Dépistage et de Traitement de la Tuberculose*) and GeneXpert molecular diagnostic platforms.

Intervention rationale: Many peripheral CDTs lacked on-site molecular testing capacity. The sample referral system aimed to link these facilities to GeneXpert-equipped laboratories through standardized sample transport mechanisms.

Pilot Phase (January to June 2023).

### 2.3. Site Selection

Twenty-nine (29) CDTs were purposively selected to represent diverse operational contexts.

#### 2.3.1. Selection Criteria

The study sites were selected to ensure representation of both urban and rural settings, including Brazzaville, Pointe-Noire, and the Cuvette department. Selection also considered the inclusion of referral facilities equipped with GeneXpert systems and peripheral sites without on-site molecular testing capacity. Only functional CDTs with ongoing TB diagnostic activities and accessible for supervision and monitoring were included. The included sites represented the maximum number that could be adequately trained, equipped, and supervised within the six-month pilot timeframe, out of 73 CDTs operating nationally in 2022; the remaining CDTs were progressively incorporated during the national roll-out phase.

#### 2.3.2. Site Categorization

For analysis, sites were categorized according to GeneXpert capacity (equipped referral sites versus peripheral referring sites) and geographic setting (urban versus rural).

### 2.4. Study Population

Patients attending participating CDTs during the pilot period; Clinical presentation consistent with presumptive pulmonary TB according to national guidelines and providing at least one sputum sample for GeneXpert testing. Where multiple samples were received from the same patient, all underwent testing, but only the initial diagnostic result was retained in case-detection counts to avoid double-counting the same patient.

### 2.5. Intervention Description

The intervention included five core components:

1. Standardized training
  - Target audience: Laboratory personnel at GeneXpert sites and staff at peripheral CDTs.
  - Content: Sputum collection techniques, biosafety procedures, triple packaging according to UN3373 standards for transport of biological substances.
  - Delivery: Standardized training sessions with dissemination of standard operating procedures and job aids.
2. Sample packaging and storage
  - Unique identifier assignment and recording in NTP registers.
  - Triple-packaging system compliant with UN3373 biosafety requirements.
  - Pre-transport storage at controlled temperature (4–8 °C).
3. Transport logistics
  - Scheduled transport: Maximum 3 rounds per week from peripheral CDTs to designated GeneXpert laboratories.
  - Transport modalities: Motorcycles (rural areas), program vehicles or contracted couriers (urban areas).
  - Cold chain maintenance: Insulated cool boxes.
  - Routes: Predefined linkages between peripheral CDTs and receiving laboratories.
4. Sample tracking system
  - Standardized tracking forms documenting each cascade step.
  - Timestamps recorded at: sample collection, dispatch, transport, laboratory receipt, testing initiation, testing completion, result validation, result communication.
  - Target timelines established to minimize diagnostic delays.
5. Laboratory procedures
  - Molecular testing platform: Xpert MTB/RIF Ultra assay (Cepheid, 904 Caribbean Drive, Sunnyvale, CA 94089, USA).
  - Testing protocol: Detection of *Mycobacterium tuberculosis* complex and rifampicin resistance per manufacturer instructions.
  - Documentation: Date and time recorded for sample receipt, test initiation, test completion, and result communication.
  - Result reporting channels: Electronic messaging (SMS/WhatsApp) as primary method, with telephone notification for positive and resistant cases.

### 2.6. Data Collection and Variables

#### 2.6.1. Operational Performance Indicators (Pilot Phase)

##### Participation Rate

✓ Definition: Proportion of selected CDTs actively transporting samples throughout the pilot period.
✓ Stratification: By geographic setting (urban vs. rural).

##### Turnaround Time (TAT)

✓ Definition of intervals measured:

Turnaround time (TAT) was assessed by measuring the time intervals across the diagnostic pathway, including the period from sample collection to dispatch at the sending CDT, dispatch to laboratory receipt, laboratory receipt to testing initiation, and testing initiation to result availability following laboratory validation. The total TAT was defined as the overall time elapsed between sample collection and result availability.

✓ Measurement method:

For samples with complete timestamp documentation, time intervals calculated between consecutive steps. Site-level referral and turnaround-time data, including timestamp completeness, are detailed in Supplementary Tables S1–S3; documentation was complete for all active sites except one (Brazzaville, *n* = 4 samples).

✓ Note on reporting approach:

Results summarized using median and range rather than proportion meeting 72 h benchmark, providing granular information about typical performance and variability.

✓ Statistical approach:

Given non-normal distributions, TAT data analyzed as medians with minimum-maximum ranges; urban and rural distributions compared descriptively.

##### Diagnostic Yield

The TB positivity rate was calculated as the number of MTB-positive samples divided by the total number of samples tested, while the rifampicin resistance rate was defined as the number of rifampicin-resistant cases divided by the total number of MTB-positive cases.

##### Operational Constraints

Operational challenges were documented through structured observations conducted during supervisory visits, incident reports submitted by participating sites, and feedback collected from healthcare staff. Identified constraints were categorized according to their nature, including logistical, administrative, technical, and communication-related issues. Their frequency and distribution were analyzed according to site characteristics such as geographic setting and activity level.

#### 2.6.2. Network Expansion Indicators (National Roll-Out Phase)

##### Geographic Coverage

Geographic coverage was assessed by measuring the number and proportion of departments participating in the sample transport system over time, with the objective of achieving nationwide implementation across all 12 departments of the Republic of Congo.

##### Network Size

The size of the diagnostic referral network was evaluated through the annual number of CDTs participating in the sample referral system and the annual number of GeneXpert machines available nationally. These indicators were compared with baseline infrastructure to assess network expansion over the study period.

##### Sample Transport Volumes

Sample transport activity was measured using the annual number of samples transported and tested through the referral system. Data were disaggregated by year and department whenever available to evaluate temporal and geographic variations in activity.

##### Diagnostic Yield over Time

Diagnostic yield was assessed through annual TB positivity rates, defined as the proportion of MTB-positive samples among all transported samples tested. The annual number of TB cases detected through the transport system, as well as the annual number of rifampicin-resistant TB cases identified, were also documented.

##### Contribution to National Case Detection

The contribution of the sample transport system to national TB case detection was evaluated by comparing the number of TB cases detected through the referral system with the total number of TB cases notified to the National Tuberculosis Program (NTP) from all diagnostic pathways. This analysis was performed separately for overall TB cases and rifampicin-resistant TB cases, with temporal trends assessed annually. Both the numerator (cases detected through the referral system) and denominator (total notified TB and RR-TB cases) were assessed at the patient level.

### 2.7. Data Sources

Primary data sources included laboratory registers from GeneXpert sites, sample transport logs, CDT patient registers, and the national tuberculosis surveillance database based on District Health Information Software 2 (DHIS2). During the pilot phase, data were collected prospectively using standardized collection forms, whereas the national roll-out phase relied on retrospective extraction from routine programmatic databases. Data quality was ensured through cross-validation between laboratory registers, transport logs, and DHIS2 entries, as well as range checks to verify the plausibility of recorded timestamps and time intervals. All patient identifiers were removed during analysis, and samples were tracked using unique laboratory codes. Baseline infrastructure indicators, including the number of CDTs and GeneXpert machines, were extracted from the 2022 National Tuberculosis Program (NTP) annual reports data for comparison purposes. National TB notification data, including figures for 2025, were validated through the routine PNLT DHIS2 quality-assurance cascade, as previously described [2].

### 2.8. Development of National Guidelines

Following completion of the pilot evaluation, the 2023 National Sample Transportation Guide was developed using pilot performance data, documentation of operational barriers, and consultations with key stakeholders including the NTP, the Ministry of Health, and implementing partners. The guidelines defined standardized operational targets, including sample dispatch within 24 h of collection with a minimum of three transport rounds per week, initiation of laboratory testing within 24 h of sample receipt, and a total turnaround time target of 72 h from sample collection to result communication. The document also established infrastructure requirements, equipment standards, training procedures, and quality assurance measures. Findings from the pilot phase, particularly regarding participation rates, turnaround time performance, and diagnostic yield, informed the decision to proceed with national scale-up and guided the development of an infrastructure expansion plan to address identified constraints.

#### National Roll-Out Phase (July 2023–December 2025)

The national roll-out phase was implemented progressively according to resource availability, transport capacity, availability of trained personnel, access to GeneXpert testing, and departmental readiness. Initial expansion in 2023 prioritized departments with existing GeneXpert capacity and established transport infrastructure, followed by intermediate expansion in 2024 as additional resources became available. By 2025, all 12 departments had been integrated into the national sample transportation system. Monitoring during this phase relied on routine programmatic surveillance through existing NTP reporting systems, with data collection focused on geographic coverage, sample transport volumes, and contribution to TB and RR case detection.

### 2.9. Statistical Analysis

Data analysis was primarily descriptive, consistent with the operational and implementation-oriented design of the study. During the pilot phase, participation rates were calculated as proportions overall and according to geographic setting. The total turnaround times were summarized using median values with minimum and maximum ranges for each diagnostic interval and for total turnaround time, while urban and rural distributions were compared descriptively without formal statistical testing. Diagnostic yield indicators were expressed as proportions and stratified by geographic setting where feasible, with 95% confidence intervals calculated using the Wilson score method. Operational constraints identified during implementation were synthesized qualitatively into thematic categories and tabulated according to site characteristics.

For the national roll-out phase, trends in network expansion (annual counts of participating CDTs and GeneXpert machines) were described using absolute and percentage changes between baseline and the end of the study period.

Annual sample transport volumes, diagnostic yield indicators, and the contribution of the referral system to national TB case detection were calculated and described over time. Data entry and cleaning were performed using Microsoft Excel, while descriptive analyses and graphical outputs were generated using GraphPad Prism version 8.0.

### 2.10. Ethical Considerations

Authorization to conduct this study was obtained from the Ministry of Health of the Republic of Congo (N°/00471/MSP/UCCP/DPNLT/22). Authorization to conduct this study was obtained from the Ministry of Health of the Republic of Congo (N°/00471/MSP/UCCP/DPNLT/22). As a programmatic evaluation using routine surveillance data conducted under the authority of the National Tuberculosis Control Program, independent ethics committee review was not required, consistent with national regulations governing the use of routine programmatic data; no individual patient identifiers were collected or analyzed. The evaluation was implemented by the National Tuberculosis Reference Laboratory under the National Tuberculosis Control Program and conducted in accordance with national public health regulations governing the use of routine programmatic data.

For any questions regarding research integrity, please contact Prof. Franck Hardain OKEMBA-OKOMBI, Director of the National Tuberculosis Control Program, franckokemba@gmail.com.

## 3. Results

### 3.1. Participation of TB Diagnostic and Referring Sites in the Pilot Evaluation

Of the 29 CDTs initially selected for the pilot phase, 25 sites (86%) actively participated throughout the evaluation period, while 4 (14%) did not.

**Figure 1:**
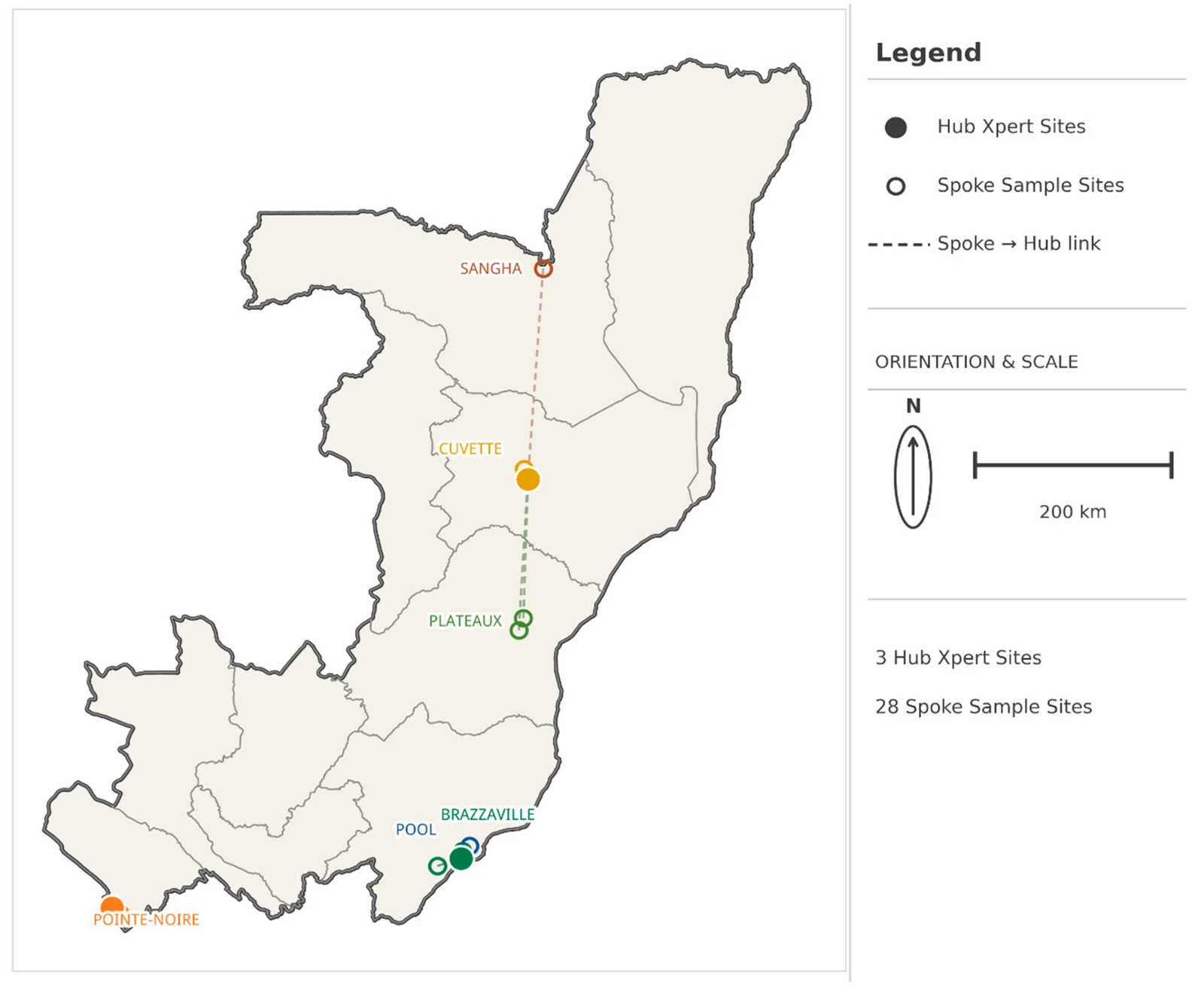
Geographic areas selected during the pilot phase of the sample transport system.

Among urban sites (Brazzaville and Pointe-Noire), 22 of 24 (92%) CDTs were active during the study period, while 2 (8%) were inactive. In rural areas (Cuvette), 3 of 5 CDTs were active (60%), whereas 2 (40%) were inactive during the same period (Table 1; Figure 1).

### 3.2. Turnaround Time Across the TB Diagnostic Cascade by Geographic Area

The median turnaround time from sample collection to result availability was 53 h (range: 19–168) in urban areas and 62 h (range: 26–204) in rural areas. The median time from sample collection to dispatch was 6 h (2–24) in urban settings and 10 h (4–36) in rural settings. The median time interval between dispatch to reception at the laboratory was 5 h (1–24) in urban areas and 10 h (6–48) in rural areas.

From sample reception at the laboratory to its testing, the median time was 18 h (4–48) in urban sites and 14 h (4–48) in rural sites. The time interval from testing to result availability had a median of 24 h (12–72) in urban areas and 28 h (12–72) in rural areas (Table 2).

### 3.3. Tuberculosis Case Detection and Drug Resistance During the Pilot Period

**Figure 2:**
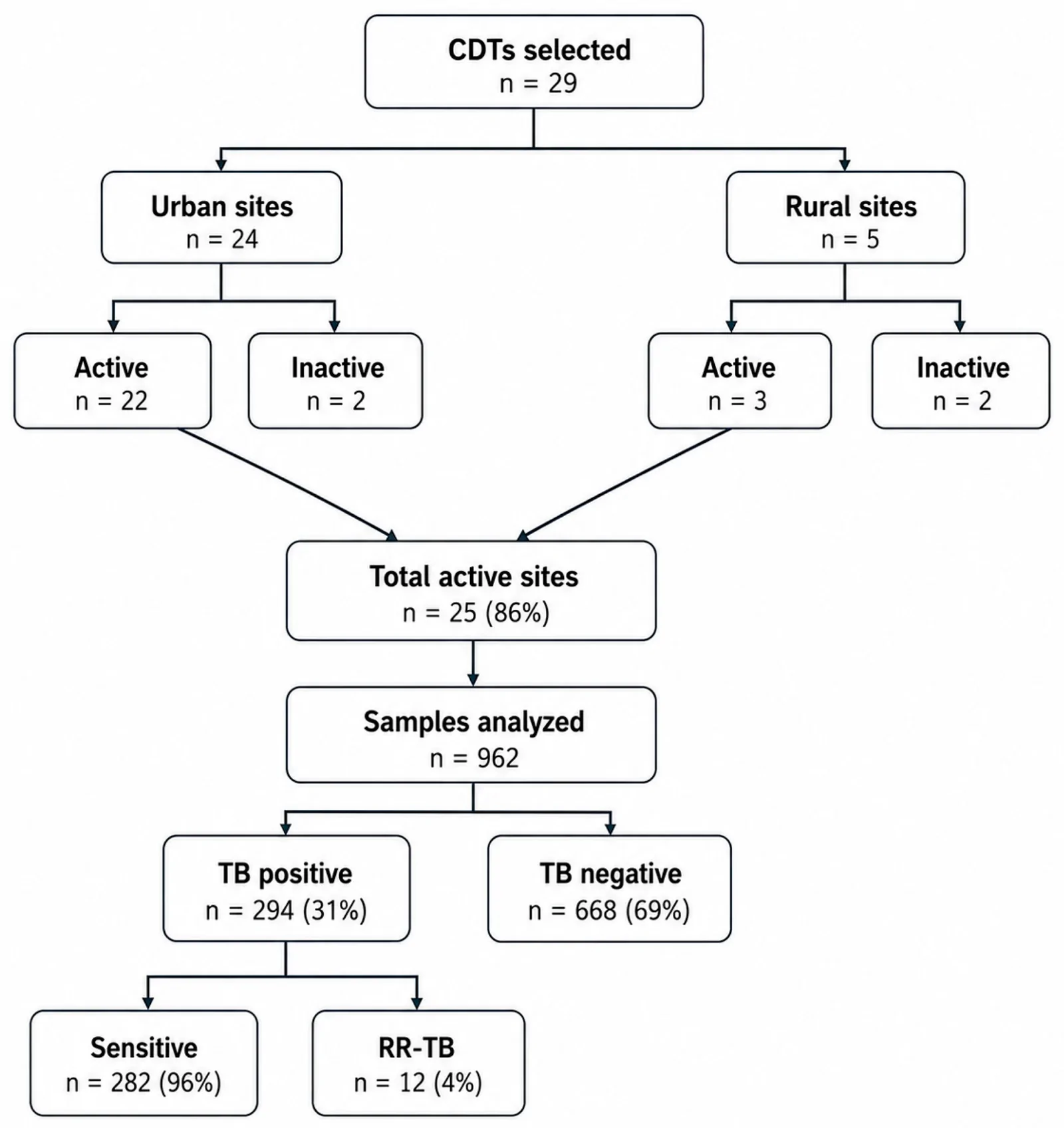
Flow Diagram Pilot Phase.

During the six-month pilot period, a total of 962 samples were transported and analyzed, leading to the detection of 294 TB cases. The urban sites (Brazzaville and Pointe-Noire) accounted for 934 (97%) transported samples and 287 detected TB cases (97.6%). Among these urban cases, 276 were drug-sensitive, while 11 were drug-resistant, yielding an overall TB detection rate of 30.7% and a resistance rate of 3.8% among positives TB cases (Table 3, Figure 2).

### 3.4. Stepwise Operational Constraints in the TB Diagnostic Workflow

During the six-month pilot, the main difficulties encountered, particularly among inactive sites (4 sites), were logistical challenges including transport constraints, lack of coolers, cold packs, and motorcycles, especially in rural areas. Other bottlenecks that weakened the activities in some centers included administrative and scheduling delays, transportation limitations, laboratory capacity constraints, and result validation/communication delays.

These bottlenecks were most pronounced in rural areas, where 2 out of 5 centers (40%) were inactive, compared to only 2 out of 24 (8%) in urban zones. All constraints and planned resolutions prior to national scale-up are summarized in Table 4.

The overall main resolution was the subsequent increase in CDT numbers across the country, from 73 in 2022 (baseline) to 113 in 2025, an increase of 54.8%, and of GeneXpert machines, from 14 in 2022 to 31 in 2025, an increase of 121.4%.

### 3.5. Establishment of the National Guidelines

Based on the rational analysis of results from the pilot phase and difficulties encountered, a pragmatic national guideline was developed: *the 2023 National Sample Transportation Guide*. Table 5 presents this guideline as a direct programmatic output of the pilot-phase findings, illustrating the translational impact of this implementation study. This table presents the standardized timeline for sample transportation, from collection to result reporting. It sets target durations for each step: dispatch within 24 h of sample collection (three times per week), testing within 24 h of reception at the laboratory, and results available at the peripheral CDT within a maximum of 72 h. This operational framework aims to ensure rapid and equitable access to molecular diagnostics, while guaranteeing sample traceability and quality throughout the country (Table 5).

### 3.6. Expansion of the Sample Transportation and Referral Network

Following the development and adoption of the 2023 National Sample Transportation Guide, the sample transportation system was progressively scaled up nationwide between 2023 and 2025 (Table 6).

The expansion was characterized not only by increased geographic coverage but also by a steady rise in the number of CDTs integrated into the referral network.

**Figure 3:**
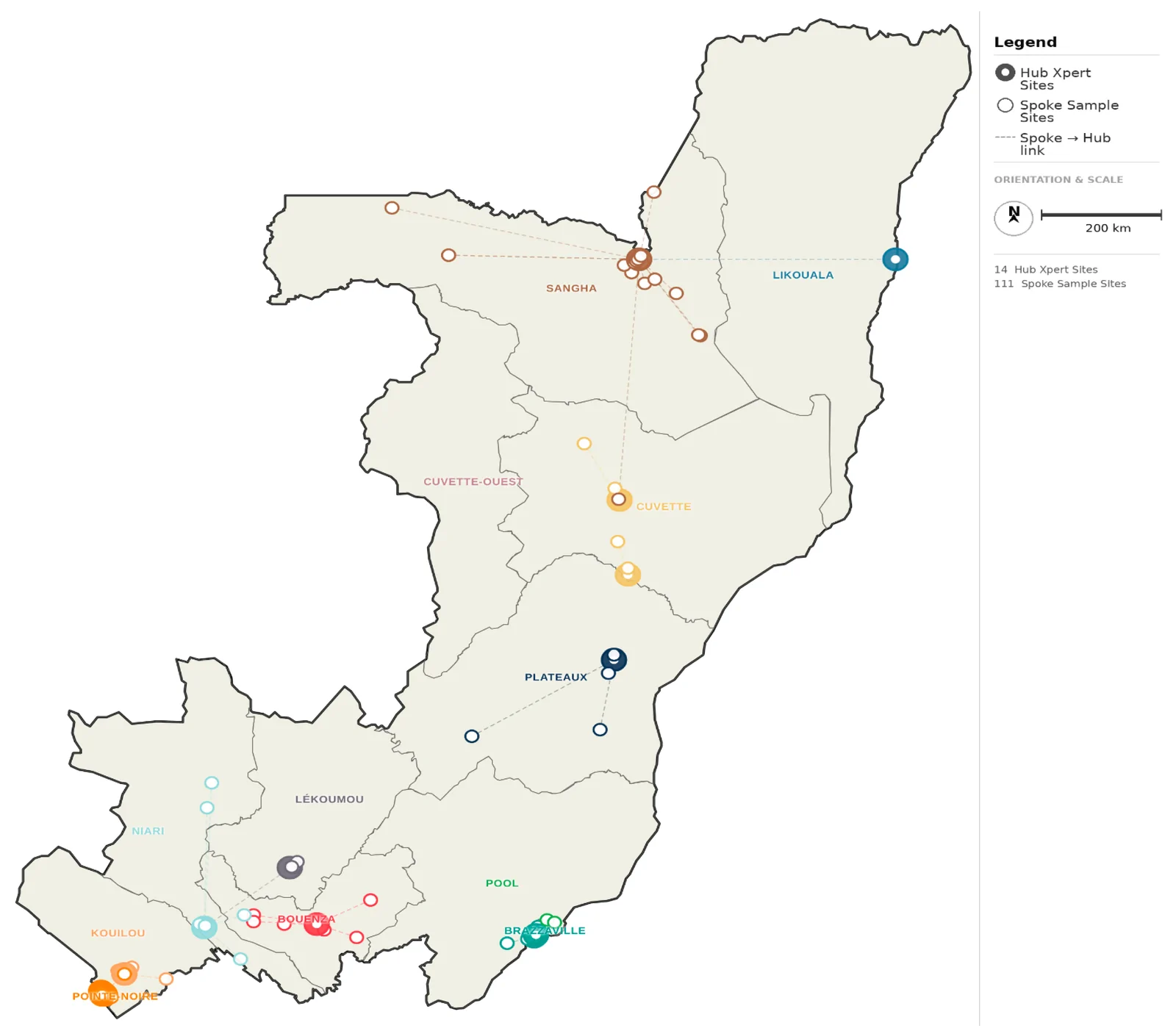
Geographic coverage of the sample transport system during the expansion Phase (by 2025).

The volume of sample transported increased to more than double between 2023 and 2024, increasing from 2154 to 5160 samples. This expansion coincided with the extension of coverage from 8 of 12 departments (67%) to 11 of 12 departments (92%), and a substantial increase in participating CDTs, from 29 to 49 facilities. In 2025, transportation volume remained high at 4861 samples while achieving full national coverage (12/12 departments). The number of CDTs involved further increased from 49 to 54, consolidating the network’s national footprint and operational capacity (Figure 3).

Over the three-year period, a total of 2587 TB cases were detected through the transport system, including 84 rifampicin-resistant cases. Annual MTB positivity rates ranged from 17.3% to 25.2%, indicating sustained diagnostic yield despite rapid scale-up.

### 3.7. Contribution of the Sample Transport System to National TB and RR Detection (2023–2025)

Between 2023 and 2025, the sample transport system demonstrated a progressive increase in its contribution to national tuberculosis (TB) detection. In 2023, 543 of 5890 nationally notified TB-positive cases were identified through the transportation network, representing 9.2% of total TB detection. In 2024, this contribution increased substantially to 894 of 7179 cases (12.5%). In 2025, although the total number of nationally reported TB-positive cases remained stable (7177), the number detected via the transport system rose further to 1150 cases, corresponding to 16.0% of national TB detection.

A similar upward trend was observed for rifampicin-resistant TB (RR-TB). In 2023, 26 of 250 nationally reported RR-TB cases (10.4%) were identified through the transport system. In 2024, 21 of 273 cases (7.7%) were detected via transport. In 2025, despite a decline in the total number of nationally reported RR-TB cases to 198, the transport system identified 37 cases, accounting for 18.7% of national RR-TB detection.

**Figure 4:**
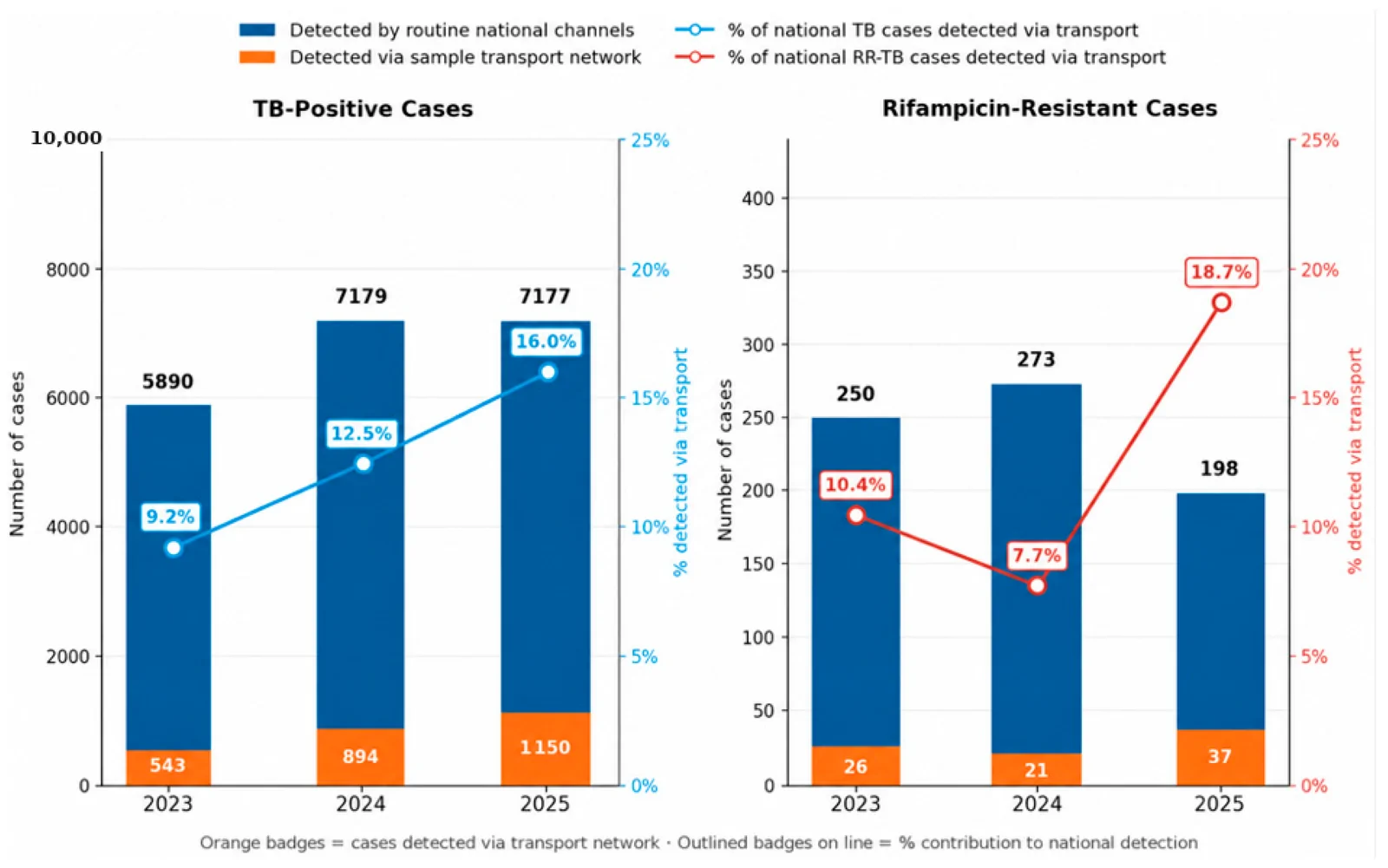
Contribution of the sample transportation system to national TB and RR detection (2023–2025).

Overall, the data indicates a steady and marked increase in the proportional contribution of the sample transportation network to both TB-positive and RR-TB case detection over the three-year period (Figure 4).

## 4. Discussion

The present evaluation suggests that the implementation of a structured sample transport and referral system is both operationally feasible and programmatically impactful within the Congolese TB diagnostic network. Similar feasibility has been reported in integrated referral pilots in Ghana and hub-and-spoke GeneXpert networks in Uganda, where structured transportation coordination improved diagnostic access in peripheral facilities [7,11]. With an overall participation rate of 86% among selected CDTs during the pilot phase, engagement in Congo compares favorably with early implementation phases reported in Uganda and Ethiopia, where initial peripheral uptake ranged between 60% and 80% [15]. The lower rural participation observed in this evaluation (60%) compared to the 92% in urban areas, mirrors disparities described in decentralized diagnostic rollouts across sub-Saharan Africa, where transportation constraints and infrastructure limitations disproportionately affect remote districts. These findings reinforce broader implementation science evidence indicating that decentralization of molecular diagnostics without proportional investment in transportation and supervisory systems risks widening geographic inequities [16,17,18,19]. It is important to note that this disparity in our setting may also reflect the relatively small number of rural centers selected for the pilot, which was partly dictated by anticipated logistical challenges.

Turnaround time (TAT) performance in Congo remained within the nationally hypothesized ≤72 h target, with median total TATs of 53 h in urban and 62 h in rural settings. Comparable studies from South Africa reported median centralized Xpert TATs between 48 and 72 h, while district-based evaluations in Uganda documented a substantial longer delay during early scale-up phases [10,20,21].

The concentration of delays in the pre-analytical phase aligns with findings from multi-country referral evaluations, which consistently identify sample collection-to-dispatch and transport intervals as the principal contributors to diagnostic delay rather than laboratory processing time. Similar patterns have been described in centralized testing networks in South Africa and integrated sample referral systems in West Africa countries. These comparisons suggest that optimization efforts in Congo should continue to prioritize route coordination, dispatch frequency and transport logistics, rather than solely expanding laboratory throughput capacity [9]. In contrast, laboratory processing time was slightly shorter in rural laboratories, likely reflecting lower testing volumes compared to high-throughput urban facilities. These overall findings suggest that optimization efforts should prioritize strengthening transport logistics, dispatch frequency, and route coordination rather than focusing exclusively on laboratory expansion [9,10,22,23].

The observed TB positivity rate of 30.6% during the pilot phase indicates appropriate clinical targeting and efficient use of molecular diagnostics. Beyond yield, the system’s contribution to national case detection increased progressively over time. For TB-positive cases, contribution rose from 9.2% of nationally notified cases in 2023 to 12.5% in 2024 and 16.0% in 2025. This represents a level of quantified national contribution rarely documented in published African evaluations, which often report site-level yield without linking results to national notification denominators. The stability of overall national TB notifications between 2024 and 2025, alongside rising absolute detection through the referral system could suggest improved diagnostic reach rather than epidemiological fluctuation alone.

The impact was even more pronounced for rifampicin-resistant TB. The proportion of nationally reported RR-TB cases detected through the transportation system increased from 10.4% in 2023 to 7.7% in 2024 and reached 18.7% in 2025. Importantly, this increase occurred despite a decline in the overall number of nationally reported RR-TB cases in 2025, suggesting that the referral system strengthened access to rapid molecular testing among populations previously underrepresented in resistance surveillance. The disproportionate growth in RR-TB contribution underscores the strategic role of organized sample transportation not only in expanding case detection but also in reinforcing national drug-resistance monitoring capacity [24].

The expansion phase between 2023 and 2025 suggests the scalability of the model. Sample transportation volumes more than doubled between 2023 and 2024, coinciding with expanded geographic coverage and increased GeneXpert deployment. The simultaneous growth in CDTs and molecular testing capacity reflects coordinated health system strengthening rather than a purely logistical intervention. The establishment of the 2023 National Sample Transport Guide provided a standardized operational framework, clarifying timelines, responsibilities, and quality assurance measures. Alignment between field implementation and national guidance likely contributed to the progressive improvement in performance indicators and detection outcomes. The concurrent growth in GeneXpert machines and CDTs likely contributed jointly with the referral system itself to rising national contribution, rather than attributing this trend to the referral system alone.

Operational bottlenecks identified during the pilot phases included transportation limitations, equipment shortages, electricity instability, and reliance on paper-based reporting reflects systemic constraints typical of resource-limited settings. Although network reinforcement addressed several barriers, post-analytical delays linked to result validation and communication remain areas for improvement. Digitalization of reporting systems and strengthened electronic connectivity between laboratories and peripheral CDTs represent critical next steps to reduce avoidable delays and ensure timely treatment initiation [25,26,27,28,29].

An additional indicator of system resilience emerged during the 2024 Mpox outbreak in the Republic of Congo. During this public health emergency, this national sample transportation network was leveraged by the Centre Opérationnel des Urgences de Santé Publique to support nationwide diagnostic response. The ability to rapidly repurpose the existing TB sample referral infrastructure for Mpox sample transportation across the national territory underscores the flexibility, scalability, and cross-programmatic utility of the model. Similar integration of TB diagnostic transportation networks into broader outbreak preparedness frameworks has been advocated by the World Health Organization as a strategy to strengthen health system resilience in resource-limited settings. This experience suggests that investments in structured sample referral systems yield benefits beyond tuberculosis control, contributing to national epidemic preparedness and emergency response capacity [30,31,32].

This programme’s achievements can also be mapped onto several WHO health system building blocks: service delivery, through extended national access to molecular testing; health information systems, through DHIS2-integrated sample tracking; medical products and technologies, through parallel expansion of GeneXpert machines and CDTs; and leadership/governance, through the 2023 National Sample Transportation Guide. This framing highlights which structural gaps, diagnostic access, data integration, and governance, were addressed, while human resources and sustainable financing remain areas for further strengthening.

Several limitations warrant consideration. The rural rifampicin-resistance rate (14.3%) was derived from a single RR-TB case among only seven MTB-positive samples and should be interpreted as descriptive only, not a stable estimate of rural resistance prevalence. The observational design precludes definitive causal attribution of improved national detection trends solely to the transportation system, as concurrent programmatic changes may have contributed. We also note the absence of economic evaluation, and that long-term sustainability, staffing, and cost implications of maintaining the network beyond the study period were not assessed. Purposive site selection does not guarantee national representativeness, and findings from this single-country implementation may not generalize to health systems with different infrastructural or organizational characteristics. Operational-constraint reporting relied on supervisory observations and staff feedback, which may underrepresent challenges at lower-performing sites, and formal fidelity metrics for SOP adherence were not implemented.

Nevertheless, the consistent upward trajectory in system contribution to both TB and RR-TB detection, particularly the marked increase in RR-TB contribution, strongly suggests meaningful programmatic impact.

Overall, these findings indicate that a structured sample transportation and referral system was associated with improvements in equitable access to molecular TB diagnosis, maintain diagnostic turnaround times within nationally defined targets, and strengthened drug-resistance surveillance. Sustained investment in transportation logistics, rural infrastructure, laboratory capacity, and digital reporting systems will be essential to consolidate these gains and ensure long-term diagnostic resilience within the national TB control strategy.

## Figures and Tables

**Table 1:** Participation of peripherals CDTs and GeneXpert Testing Sites by Geographic Area.

Area	GeneXpert Sites	Sending Sites (CDT) Involved	Active Sending Sites	Inactive Sending Sites	Active Participation Rate (95% CI)
Urban (Brazzaville + Pointe-Noire)	3	24	22	2	92% (74.2–97.7)
Rural (Cuvette)	1	5	3	2	60% (23.1–88.2)
TOTAL	4	29	25	4	86% (69.4–94.5)

**Table 2:** Median turnaround time (h) by process step and geographic area.

Process Step	Area	Median (h)	Min–Max (h)
From collection to dispatch	Urban	6	(2–24)
	Rural	10	(4–36)
From dispatch to Reception at the laboratory	Urban	5	(1–24)
	Rural	10	(6–48)
From reception at the laboratory to testing	Urban	18	(4–48)
	Rural	14	(4–48)
From sample testing to availability of results	Urban	24	(12–72)
	Rural	28	(12–72)
TOTAL (From sample collection to availability of result)	Urban	53	(19–168)
	Rural	62	(26–204)

**Table 3:** Distribution of samples, TB detection yield, and drug resistance by geographic area. Pilot phase (January–June 2023).

Zone	Samples	MTB+	DS TB	DR TB	TB Detection Rate (95% CI)	RR Rate (95% CI)
**Urban**	934	287	276	11	30.7% (27.9–33.8)	3.8% (2.2–6.7)
**Rural**	28	7	6	1	25% (12.7–43.4)	14.3% (2.6–51.3)
**Total**	962	294	282	12	30.6% (27.7–33.5)	4.1%(2.4–7.0)

MTB+: Mycobacterium tuberculosis-positive; DS TB: drug-sensitive tuberculosis; DR TB: drug-resistant (rifampicin-resistant) tuberculosis.

**Table 4:** Operational constraints and planned resolutions across the TB diagnostic cascade.

Process Step	Major Operational Constraints	Observed Challenges	Resulting Programmatic Action
From collection to dispatch	Administrative and scheduling delays	Staff workload; fixed pick-up schedules; accumulation of samples before dispatch	Reinforce staff training on sample preparation; emphasize importance of dedicated pick-ups; implement staggered dispatch schedules
From dispatch to laboratory reception	Transportation limitations	Long distances; poor road conditions (especially in rural zones)	Increase number of Xpert testing sites; optimize transport routes; allocate motorcycles and fuel for sample transportation
From laboratory reception to testing	Laboratory capacity limitations	High testing volume vs. Xpert MTB/RIF Ultra capacity; electricity interruptions	Expand laboratory capacity; maintain buffer stock of Xpert MTB/RIF Ultra cartridges; regular equipment maintenance; equip Xpert sites with backup power (generator or solar)
From testing to result availability	Result validation and communication delays	Paper-based reporting; delayed verification; lack of electronic connectivity; delayed clinician notification	Implement electronic reporting; establish SMS/WhatsApp alerts for clinicians; integrate laboratory-facility communication platform

**Table 5:** Optimized national timeline (Collection to result).

Process Step	Description	Responsible Party	Frequency/Timing	Target Duration
1. Collection	Sputum sample collected from presumptive TB case at peripheral CDT	Peripheral CDT	Continuous	N/A
2. Packaging	Triple-layer packaging with cold packs	Sending Site Staff	Before each shipment	N/A
3. Dispatch	Sample dispatched from peripheral CDT to receiving GeneXpert site	Sending Site Staff	Monday, Wednesday, Friday (by 12:00 p.m.)	Within 24 h of collection
4. Transport (Intra-Urban)	Sample transported by vehicle or moto	Transporter (Lab staff)	Same day as dispatch	≤2–4 h
5. Transport (Inter-Departmental)	Sample transported by bus or vehicle; sender informs receiver of arrival time	Transporter (Lab staff)	As scheduled	Variable (same-day priority)
6. Lab receipt and verification	Sample logged, checked for integrity, temperature, and documentation	Receiving Lab Staff	Immediate upon arrival	≤15 min
7. Testing	Xpert MTB/RIF Ultra assay performed	Receiving Lab staff	Upon receipt	≤24 h of receipt
8. Result Reporting	Results communicated back to referring CDT	Receiving Lab Staff	After validation	≤24 h (target)
9. Result Available at CDT	Results received by peripheral CDT for patient notification and care initiation	Sending Site Staff	Per retrieval schedule	≤72 h (max) from dispatch
TOTAL (From collection to result)	End-to-end timeline	All parties	N/A	≤72 h (48–72 h target)

**Table 6:** Coverage and scale-up over time (2023–2025).

Year	Total Samples Transported	MTB-Positive *n* (%)	Rifampicin-Resistant *n* (%)	% Departments Covered *n* (%)	Number of CDT Involved
**2023**	2154	543 (25.2%)	26 (4.8%)	8/12 (67%)	29
**2024**	5160	894 (17.3%)	21 (2.3%)	11/12 (92%)	49
**2025**	4861	1150 (23.7%)	37 (3.2%)	12/12 (100%)	54
**Total**	12,175	2587 (21.2%)	84 (3.2%)	12 (100%)	54

## Data Availability

The datasets generated and/or analyzed during the current study are not publicly available due to national health data protection regulations but are available from the corresponding author on reasonable request and with permission from the National Tuberculosis Control Program of the Republic of Congo.

## Supplementary Materials

The following supporting information can be downloaded at: https://www.mdpi.com/article/10.3390/tropicalmed11080229/s1, Table S1: Sample referrals to CAT-Brazzaville GeneXpert Site by Referring Site; Table S2: Sample referrals to CAT-Pointe-Noire and Tié-Tié Hospital GeneXpert Site by Referring Site; Table S3: Sample referrals to July 31 Hospital GeneXpert Site by Referring Site.
