# Supplementary material for: National Implementation of a Tuberculosis Sample Referral Network to Expand Access to Molecular Diagnosis in the Republic of Congo: Pilot Evaluation and Programmatic Analysis (2023–2025)
Source: Trop Med Infect Dis. 2026 Aug 17;11(8):229. doi: 10.3390/tropicalmed11080229 (PMC13517841; doi:10.3390/tropicalmed11080229)
Supplement: Supplementary file 1 [file tropicalmed-11-00229-s001.zip › tropicalmed-4413734-supplementary.pdf]

Supplementary Table S1: Sample referrals to CAT-Brazzaville GeneXpert Site by Referring Site

Referring Site	Samples Referred	TB Detected	RR Detected	Status Throughout Study	Median Turnaround Time (hours)
**Site 1**	85	33	0	Active	31h
**Site 2**	0	0	0	**Inactive**	NA
**Site 3**	32	5	0	Active	55h
**Site 4**	15	1	0	Active	72h
**Site 5**	24	1	1	Active	87h
**Site 6**	37	23	0	Active	28h
**Site 7**	133	75	3	Active	30h
**Site 8**	5	1	0	Active	53h
**Site 9**	4	0	0	Active	NA
**Site 10**	11	1	0	Active	168h
**Site 11**	18	1	0	Active	112h
**Site 12**	1	0	0	Active	19h
**Total Brazzaville**	366	141	4		58h

Supplementary Table S2: Sample referrals to CAT-Pointe-Noire and Tié-Tié Hospital GeneXpert Site by Referring Site

Referring Site	Samples Referred	TB Detected	RR Detected	Status Throughout Study	Median Turnaround Time (hours)
Site 1	18	4	0	Active	20h
Site 2	30	4	1	Active	101
Site 3	31	8	0	Active	50h
Site 4	123	39	4	Active	80h
Site 5	152	32	1	Active	25h
Site 6	22	7	0	Active	22h
Site 7	85	16	1	Active	36h
Site 8	57	9	0	Active	37h
Site 9	5	0	0	Active	100h
Site 10	6	3	0	Active	68h
Site 11	0	0	0	Inactive	92h
Site 12	56	12	0	Active	40h
Total Pointe-Noire	568	135	7	11 active / 1 inactive	50h

Supplementary Table S3: Sample referrals to *July 31 Hospital* GeneXpert Site by Referring Site

**Referring Site**	**Samples Referred**	**TB Detected**	**RR Detected**	**Status Throughout Study**	**Median Turnaround Time (hours)**
**Site 1**	1	0	1	Active	26h
**Site 2**	0	0	0	**Inactive**	**NA**
**Site 3**	5	2	0	Active	204h
**Site 4**	0	0	0	**Inactive**	NA
**Site 5**	22	4	0	Active	62h
**Total Cuvette**	**28**	**6**	**1**	3 active / 2 inacttives	62h
